# A Comparison of Emotionally Stimulated and Conventionally Collected Tears Using Bottom‐Up, Label‐Free Quantitative Proteomic Analysis—A Pilot Study

**DOI:** 10.1002/prca.70023

**Published:** 2025-09-18

**Authors:** Campbell Bruce Mousseau, Alena R. Veigl‐Lunsford, Rhonda L. Pitsch, Sean W. Harshman

**Affiliations:** ^1^ AV Inc. Air Force Research Laboratory 711th Human Performance Wing/RHBBA Wright‐Patterson AFB Ohio USA; ^2^ Air Force Research Laboratory 711th Human Performance Wing/RHBBA Wright‐Patterson AFB Ohio USA

**Keywords:** body fluids, bottom‐up proteomics, comparative proteomics, electrospray ionization mass spectrometry (ESI‐MS), label‐free quantification, mass spectrometry ‐ LC‐MS/MS

## Abstract

Proteomic analysis of biofluids is central for identifying disease biomarkers. Tears have become popular targets for biomarker discovery and biosensor development, largely because they can be collected noninvasively and are rich sources of biomarkers for ocular and systemic diseases. Although basal and reflex tears have been well characterized, the proteome of psycho‐emotionally stimulated tears remains largely unexplored, hindering their applicability in biomarker discovery studies and the advancement of tear‐based biosensors. Comprehensive proteomic analysis across different tear types is crucial for identifying novel biomarkers and improving disease diagnosis and monitoring. In this pilot study, tears collected via conventional stimulation techniques (“standard”) versus those elicited through emotional stimuli (“emotional”) were purchased from single donors through two vendors. We compared the proteomic profiles of emotional (*n* = 6) and standard (*n* = 14) single donor human tears to better understand the biochemical composition and functional roles of different tear types. In total, 907 proteins were identified from all tear samples. Fifty‐two tear proteins were significantly enriched in emotionally stimulated tears. Functional characterization of enriched proteins revealed that most were extracellular or secreted. Many were also involved in host defense or immune responses, including members of the S100A and neutrophil defensin protein families.

**Summary:**

- Although emotional tears are known to differ from basal and reflex tears in both composition and function, the specific biochemical characteristics and functional roles of emotional tears remain poorly understood.
- This gap in knowledge is largely due to the limited research conducted on emotional tears, despite their distinct origins.
- A more complete understanding of all tear types is necessary for the continuation of biomarker discovery and the development of tear fluid‐based biosensors.
- In this exploratory study, tears collected via a conventional/standard protocol and those collected from emotional stimulus were obtained from two vendors and subjected to proteomic profiling and comparison.
- A bottom‐up proteomic approach was utilized to analyze tear samples, facilitating a comparison between tear types and contributing to the characterization of psycho‐emotional tears.

**Abbreviations**
AMP: antimicrobial peptide
BCA: bicinchoninic acid
B‐H: Benjamini–Hochberg
CV: coefficient of variation
DDA: data‐dependent analysis
DED: dry eye disease
FA: formic acid
FC: fold change
GO: Gene Ontology
HLB: hydrophilic–lipophilic balance
LFC: Log_2_(fold change)
LFQ: label‐free quantification
LPS: lipopolysaccharide
PANTHER: Protein Analysis Through Evolutionary Relationships
PD: Proteome Discoverer
sEV: small extracellular vesicle
S‐Trap: suspension trapping
STRING: Search Tool for the Retrieval of Interacting Genes/Proteins

## 1 Introduction

The ocular surface is protected by a constitutively produced tear film that provides lubrication, nutrients, and defense against pathogens and environmental debris [1]. Human tears are rich in proteins and offer valuable biomarkers for ocular and systemic diseases, while presenting a less complex matrix than serum or plasma [2, 3, 4]. Growing interest in tear analysis for biomarker discovery and biosensor development stems from the ease with which tears can be collected and the tear film's reflection of surrounding tissue physiology [5]. Due to this, tear‐based diagnostic studies have expanded beyond just ocular diseases like glaucoma and Sjögren's syndrome. Recent publications utilized tears to identify differentially expressed proteins in patients with diabetes, Parkinson's disease, and multiple forms of cancer [6, 7, 8, 9]. Wearable contact and eye patch biosensors have been developed with the ability to monitor these and many other biomarkers to improve personal health [10, 11].

Tear proteomics holds significant potential as a noninvasive diagnostic tool for assessing a patient's well‐being. Advancements in liquid chromatography‐tandem mass spectrometry (LC‐MS/MS) technologies have significantly improved biomarker analysis of biofluids for health care monitoring [12, 13]. For tears, it is no longer necessary to pool samples for analysis, as superior MS instrumentation enables the accurate identification of thousands of proteins from microliter volumes of individual samples [14]. This and the in‐depth detection of differentially expressed proteins, combined with systems biology analysis, provide candidate biomarkers of clinical relevance and insights into the subtle compositional changes linked to disease onset, progression, or treatment response [15, 16, 17]. For example, studies evaluating the composition of tears have shown that proteins MMP‐9 and lipocalin‐1 are commonly linked to symptoms of dry eye disease (DED) [18]. Another study identified 14 proteins that were differentially expressed in the tears of breast cancer patients from <10 µg of starting protein material [9]. Further biomarker discovery and monitoring studies using tears are essential for developing treatments for these and other conditions.

Humans produce three types of lacrimal tears: basal, reflex, and psycho‐emotional (emotional) tears [19, 20]. The function and mechanism of basal and reflex tears are well known; however, emotional tears are rarely studied and are therefore poorly understood [21]. This represents a significant obstacle for biomarker analysis, and more recently, biosensor development, as studies show that tear composition is highly dynamic and dependent on the specific tear type [22, 23]. Further investigation into the composition of all tear types could benefit the development of ocular biosensors aimed at improving the timely diagnosis and treatment of medical conditions [24, 25]. To this end, we purchased 20 single‐donor tear samples collected using conventional means (standard) or through emotional stimulation, and performed bottom‐up proteomic analyses to compare the two. This analysis was performed to understand how emotional stress might influence tear composition, which could elevate the capacity to evaluate broader physiological and psychological disorders and improve tear‐based wearable biosensors, which are often challenged by variations of tear composition due to emotional or mechanical stimuli [26].

## 2 Materials and Methods

### 2.1 Tear Collection and Vendor Information

Fourteen single‐donor standard human tears were acquired in 100 µL aliquots through Medix Biochemica (Uusimaa, FIN) or Innovative Research (Novi, MI) (eleven female, three male, average age of 30.07 ± 11.77 years, all Caucasian). Six emotional 100 µL single donor tear samples were purchased from Medix Biochemica (five female, one male, average age of 37.33 ± 15.08 years, four Caucasian, one Asian, and one Asian/Caucasian). This represented the maximum available quantity for each lot number from both samples. Subject and vendor information for each sample is provided in Table 1. In the standard collection protocol, tears were collected directly into a vial after drying the subject's eyes using air pressure or by manually holding the eyelids open. Emotional tears were collected passively into a vial without any external stimulation. Each single‐donor sample (*n* = 20) was categorized as either “emotional” or “standard” depending on collection type. The 20 tear samples were prepared, without pooling, for bottom‐up proteomic analysis as described below.

**TABLE 1:** Vendor and subject information.

Donor	Vendor^a^	Age	Race^b^	Gender	Emotional (Y/N)
T1	MB	19	A	F	Y
T2	IR	57	C	F	N
T3	IR	19	C	F	N
T4	IR	23	C	F	N
T5	MB	24	C	M	N
T6	MB	28	C	F	N
T7	MB	56	C	F	Y
T8	IR	19	C	F	N
T9	IR	29	C	F	N
T10	MB	44	C	M	Y
T11	IR	37	C	F	N
T12	MB	30	C	M	N
T13	MB	19	C	F	N
T14	IR	31	C	F	N
T15	IR	24	C	M	N
T16	MB	23	A/C	F	Y
T17	MB	53	C	F	Y
T18	MB	29	C	F	Y
T19	IR	53	C	F	N
T20	IR	28	C	F	N

^a^ MB denotes Medix Biochemica and IR denotes Innovative Research.

^b^ C denotes Caucasian, A denotes Asian, and A/C denotes Asian and Caucasian.

### 2.2 Bottom‐Up Proteomics Sample Preparation

Tears were clarified by centrifugation at 10,000 rcf for 10 min at room temperature to remove cellular debris, and protein concentration was determined via bicinchoninic acid (BCA) assay (Thermo Scientific, San Jose, CA) with BSA as a standard using a microplate reader (BioTek Synergy 2, Winooski, VT). Protein concentrations for each sample are provided in Table S1. For each sample, 50 µg aliquots were removed and concentrated to near dryness in a vacuum centrifuge (SP Genevac miVac, Warminster, PA) and prepared for bottom‐up proteomic analysis as described previously [27]. Briefly, dried samples were resuspended in buffered sodium dodecyl sulfate (SDS, 7%), reduced with 10 mM tris(2‐carboxyethyl)phosphine (TCEP, Sigma–Aldrich, St. Louis, MO) at 60°C while shaking at 1000 rcf for 20 min in a thermocycler C (Eppendorf, Hamburg, GER), then alkylated in the dark for 30 min with 10 mM iodoacetamide (IAA, Sigma–Aldrich). Samples were filtered and digested using suspension trappings (S‐Traps, Protifi, Huntington, NY) following the manufacturer's instructions [28]. The resulting peptides were concentrated to near dryness after elution.

### 2.3 Solid Phase Extraction (SPE)

Peptides were resuspended in 0.1% formic acid (FA, Thermo Fisher Scientific) before desalting. All samples were desalted before analysis with 10 mg hydrophilic–lipophilic balance (HLB) SPE cartridges (Waters Corporation, Milford, MA) as in Mousseau et al. and Cronin et al., with slight modifications [29, 30]. Briefly, SPE cartridges were equilibrated with three 300 µL washes with acetonitrile (ACN, Sigma–Aldrich) followed by three 300 µL washes with 0.1% FA. Samples were passed through the cartridges using positive pressure and washed with 0.1% FA. Cleaned peptides were eluted in 50% ACN with 0.1% FA, then dried in a vacuum centrifuge.

### 2.4 Analysis via Nano‐Flow HPLC‐MS/MS

Dried peptides were resuspended in 0.1% FA to a concentration of 500 ng/µL. For analysis, 1 µg was separated following a trap‐and‐elute workflow as described in previous publications with minor alterations [31, 32]. A 5 mm x 300 µm C_18_ PepMap Neo Trap Cartridge (Thermo Fisher Scientific) was washed with 0.03% trifluoroacetic acid (TFA, Sigma–Aldrich) in 2% ACN at 5 µL/min for 7.5 min before aliquots of samples were injected onto the trap column to desalt and concentrate peptides. After desalting, peptides were injected onto an EASY‐Spray 100 mm x 75 µm C_18_ reversed‐phase column on an RSLC Nano (Ultimate 3000, Thermo Fisher Scientific). Peptides were separated at 0.3 µL/min over a 77.5‐min, segmented gradient from 2% to 90% *B* (*A* = 0.1% FA, *B* = 0.1% FA in ACN). MS‐analysis was performed on a Thermo Scientific Orbitrap Fusion Lumos Tribrid Mass Spectrometer at a resolution of 120,000 in positive ion mode. Effluents were introduced into the mass spectrometer via nano‐electrospray ionization (ESI) using a Thermo Fisher EASY‐Spray source heating the column at 35°C with a spray voltage of 2.3 kV. Data was acquired in technical triplicate using data‐dependent analysis (DDA). Charge States 2–7 were included at an intensity threshold of 1.0E + 04 with 3 s between master scans. Collision‐induced dissociation (CID) was performed with the activation energy fixed at 35% for an activation time of 10 ms. A more comprehensive summary of the separation and MS methods can be found in the Supporting Information.

### 2.5 Protein Identification

Data files from technical and biological triplicates were searched against the UniProt database of *Homo sapiens* (downloaded March 16, 2023) using Proteome Discoverer (PD) v2.5 for label‐free quantification (LFQ) as in Xu et al., with minor modifications [33]. “Emotional” and “Standard” were used as categorical factors, and sample numbers were used as biological factors. Files were searched with Trypsin designated as the enzyme, selectively permitting up to two missed cleavages using Sequest HT for spectrum matching. Oxidation of methionine was selected as a dynamic modification, and carbamidomethylation of cysteine residues was set as a static modification with a precursor mass tolerance of 10 ppm and a fragment mass tolerance of 0.6 ppm. A maximum of four dynamic modifications were allowed per peptide with a minimum length and charge of six amino acids and +2, respectively. For spectrum matching, b and y ions were used, and the minimum and maximum precursor masses were set to 350 and 5000 Da, respectively. Peptide spectral matching (PSM) and protein identifications were filtered to a strict false discovery rate (FDR) of 0.01 and a relaxed FDR of 0.05 using the percolator node. Unique and razor peptides were used for general quantification, and precursor abundance was based on intensity. All peptides were used for normalization and protein roll‐up; modified peptides were excluded for pairwise ratios. Summed abundances were used for protein abundance calculations.

### 2.6 Proteomics Data Analysis

For all proteomic data, the output generated by the PD v2.5 software was statistically analyzed using Microsoft Excel for Microsoft 365 MSO and R Studio (v4.3.1) (Tables S2 and S3). A Student's *t* test was used to identify significant differences among emotional and standard sample groups at an FDR of 0.01. The resulting *p* values were adjusted using the Benjamini–Hochberg (B‐H) method to determine a new significance threshold [34]. After B‐H correction, differences for which *p* was <0.027 (or −Log_10_(*p*) > 1.57) were considered significant (Table S5). To determine the fold change (FC) values for identified proteins, their average measured abundance in emotional tear samples was divided by their corresponding average abundance in standard tear samples (emotional/standard). Log_2_(FC) values (LFC) > |1.2| and *p* values < 0.027 were used as screening criteria for the selection of significantly enriched proteins.

### 2.7 Bioinformatic Analysis

To characterize protein interactions, the Search Tool for the Retrieval of Interacting Genes/Proteins (STRING, v12.0, https://string‐db.org/) database was used to illustrate the interconnectivity of significantly enriched proteins [35]. Protein lists were analyzed using *H. sapiens* as the organism at an FDR of 5%.

## 3 Results

### 3.1 Sample Preparation and Analysis

**FIGURE 1:**
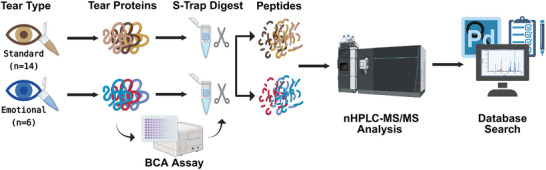
Experimental design: Tears collected after emotional (*n* = 6) or conventional/standard stimulus (*n* = 14) were sourced from two vendors and prepared for bottom‐up proteomic analysis. Proteins were extracted and enzymatically digested into peptides before separation and analysis via reverse‐phase nano‐flow HPLC‐MS/MS. The resulting data files underwent a spectral library search and statistical analysis using Proteome Discoverer—figure generated with www.Biorender.com.

Emotional tears represent a relatively understudied class of biofluids [20]. The limited research available leaves many questions about their specific roles and their underlying mechanisms unanswered [21]. Using the workflow outlined in Figure 1, global proteome changes in tear film postemotional stimulation were assessed and compared to conventionally collected tears [36]. The concentration of each tear sample was taken via BCA assay, and protein concentration was significantly higher (*p* < 0.05), on average, in emotional tears compared to standard tears (Figure S1). This agrees with previously reported data [37, 38].

**FIGURE 2:**
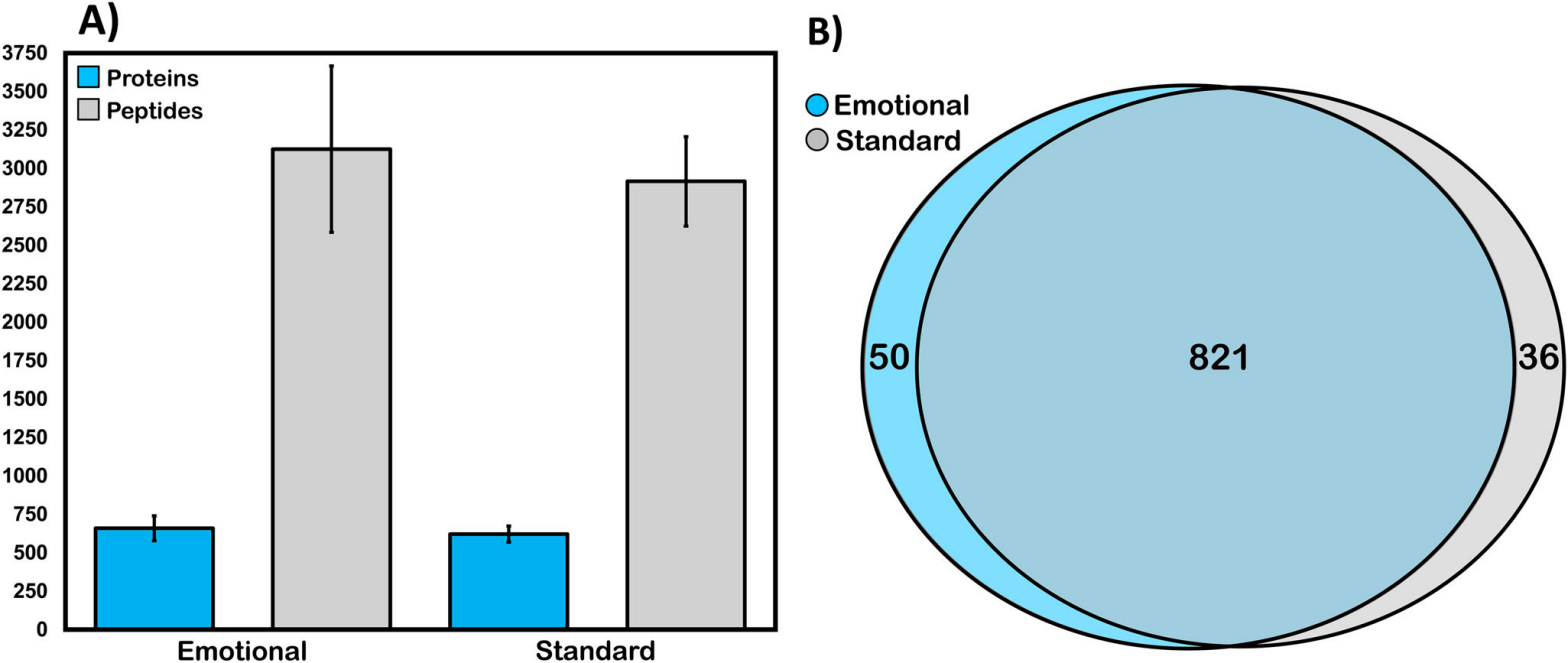
(A) A bar graph showing the average number of protein and peptide identifications (error bars = standard deviation) for either tear categorical factor (emotional or standard) per biological replicate and (B) a Venn diagram illustrating common and unique proteins from the total number of proteins identified. There is a large overlap in protein identifications between the two sample types.

After MS analysis, a total of 907 proteins and 4863 peptides were identified in all 20 samples. On average, 659.50 (±80.87) proteins and 3125.5 (±541.17) peptides were identified per biological replicate in emotional tears, and 621.71 (±52.53) proteins and 2915.86 (±290.92) peptides were identified per biological replicate in standard tears (Figure 2A). In total, 871 proteins and 4558 peptides were identified in emotional tears, and 857 proteins and 4419 peptides were identified in standard, nonstimulated tears. Of the proteins identified, 50 were unique to emotional tears and 36 were unique to standard tears. Overall, the proteomic composition of standard and emotional tears was largely similar, with more than 90% of all proteins identified being found in both sample types (Figure 2B). Furthermore, each sample's Top 20 most abundant proteins remained consistent, and 90 of the Top 100 most abundant proteins were also the same between sample types (Table S4).

**FIGURE 3:**
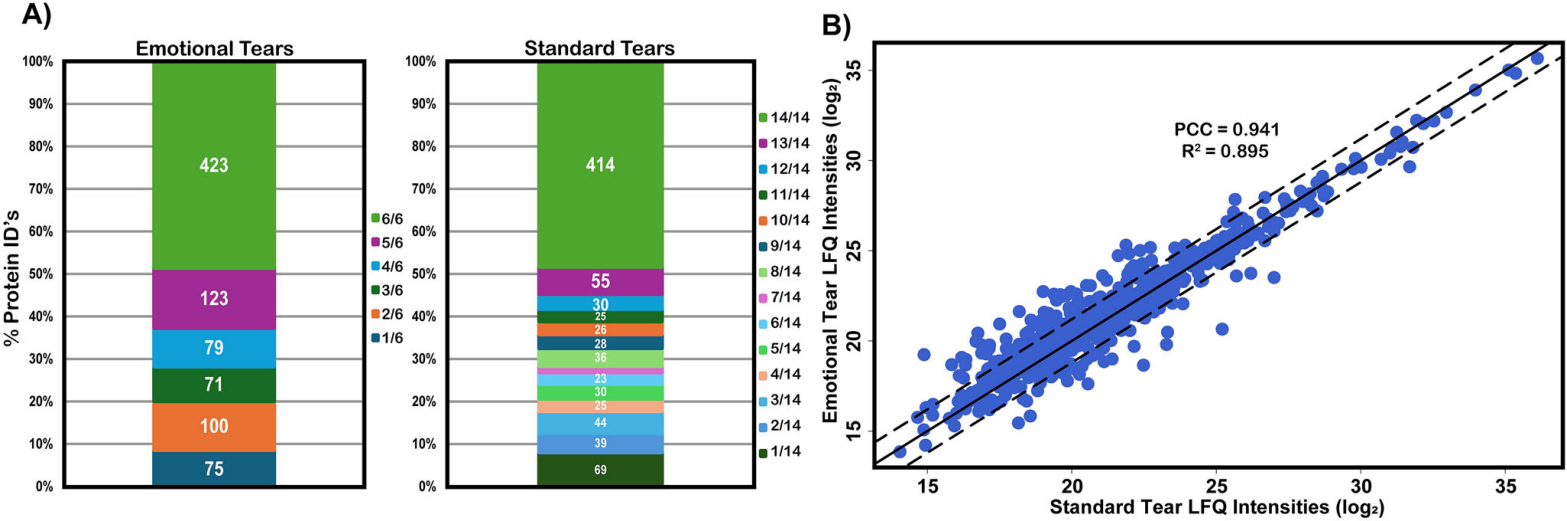
(A) Stacked bar graphs illustrating the percent of total proteins identified by sample type per biological replicate. The colored boxes represent the number and percentage of proteins identified as well as the frequency for each sample type. The data show that approximately 80% of all proteins were identified in at least half of the biological replicates and 50% were identified in all biological replicates. (B) The average log_2_‐abundance values of proteins from each sample type are plotted against each other. Points within the dashed lines and along the solid line indicate a log_2_‐fold change (LFC) of less than 1.2. Points outside the dashed lines have absolute LFC values greater than 1.2 (LFC > |1.2|). There are 709 proteins within the dashed lines and 112 outside; however, there is a strong linear correlation between the sample types (Pearson correlation coefficient = 0.941).

A stacked bar graph was created to determine the frequency in which proteins were identified by sample type (Figure 3A). For both emotional and standard tears, 80% of proteins identified were present in at least half of the biological replicates and ∼50% of proteins identified were present in all biological replicates. These findings are similar to a previous study, which reported an overlap in 45.5% of the proteins identified from three different tear samples pooled from multiple subjects [39].

Next, protein abundances were averaged and converted to log_2_(abundance) to create a scatter plot with standard tear abundances on the *x*‐axis and emotional tear abundances on the *y*‐axis (Figure 3B). The solid line going through the scatter plot indicates where the average protein abundance is roughly the same (LFC < 1.2). Points outside the two dashed lines have absolute LFC values greater than 1.2. The relationship between average protein abundance for either sample type showed a strong linear correlation (PCC = 0.941). Most (709) identified proteins had similar abundance ratios.

### 3.2 Comparison to Other “Tear Proteomics” studies

**FIGURE 4:**
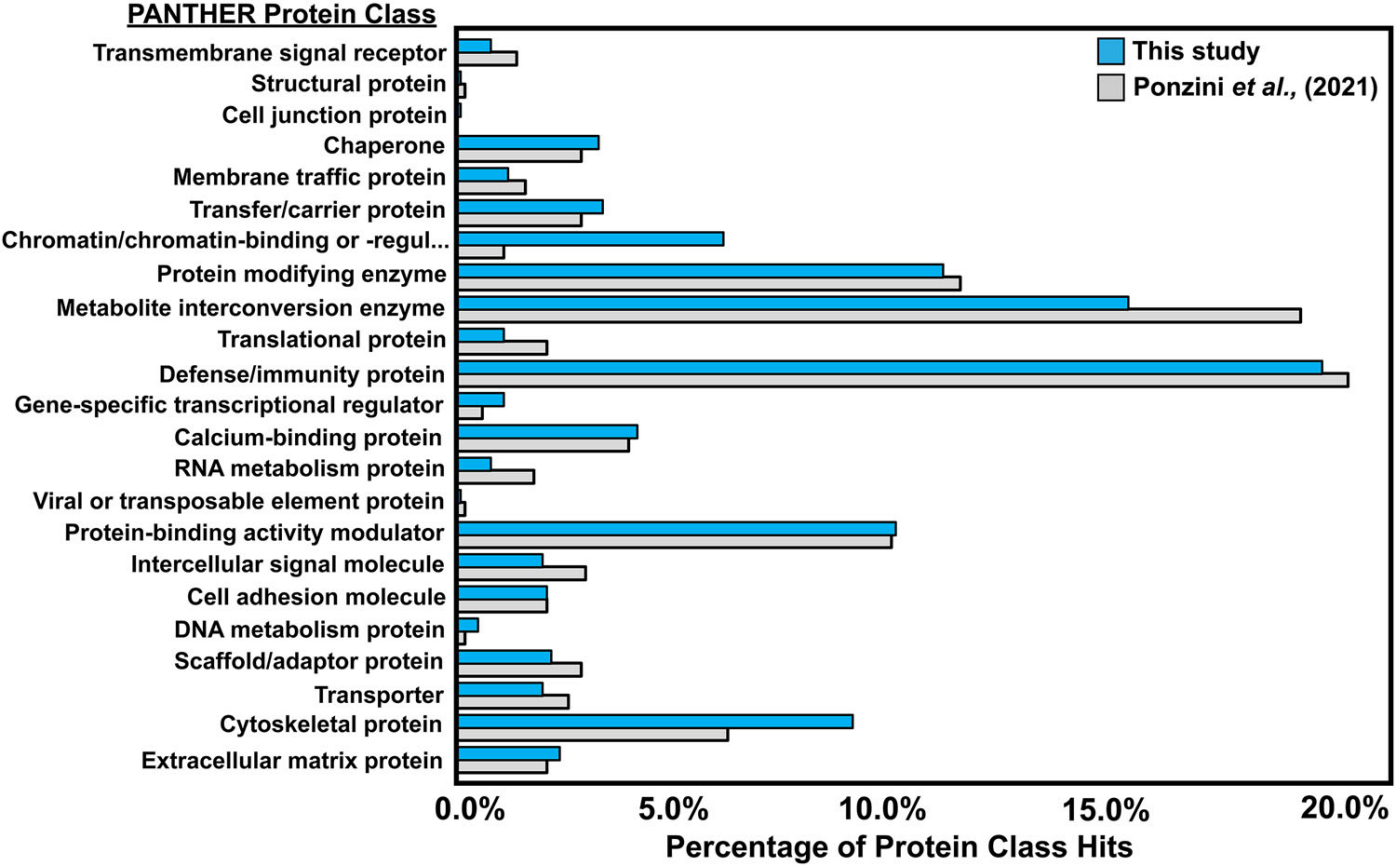
A bar plot comparing the percentage of hits for each protein class in this study versus those from a study conducted by Ponzini et al. [41]. The identified protein classes and distributions are similar between this study and the Ponzini et al. study.

The identified proteins were categorized by class using the functional annotation clustering tool from Protein Analysis Through Evolutionary Relationships (PANTHER) [40]. The same analysis was performed on the protein list reported by Ponzini et al. to compare this work with theirs [41]. In both this study and that reported by Ponzini et al., the protein classes of metabolite interconversion enzyme, defense/immunity protein, protein‐modifying enzyme, and protein‐binding activity modulator received the most hits (Figure 4). Overall, classes identified using PANTHER analysis were consistent across both studies and had similar distributions.

The PANTHER functional annotation clustering tool was again used to compare the molecular functions of proteins from this study with those reported by Tse et al., who also utilized S‐Traps to digest tear proteins compared to an in‐solution protocol (Figure S2) [42]. In both this study and that from Tse et al., binding and catalytic activity account for more than three‐quarters of the total functional hits. For the S‐Trap method, the distribution of molecular functions is largely the same between the studies, except for “antioxidant activity,” which was not reported previously, but represents less than 2% of the total class hits. These findings confirm that our results align with previously published studies. The consistency in identifications, class, and function suggests that core functional proteins are robustly conserved, regardless of stimulation.

### 3.3 Variability Between Replicates

**FIGURE 5:**
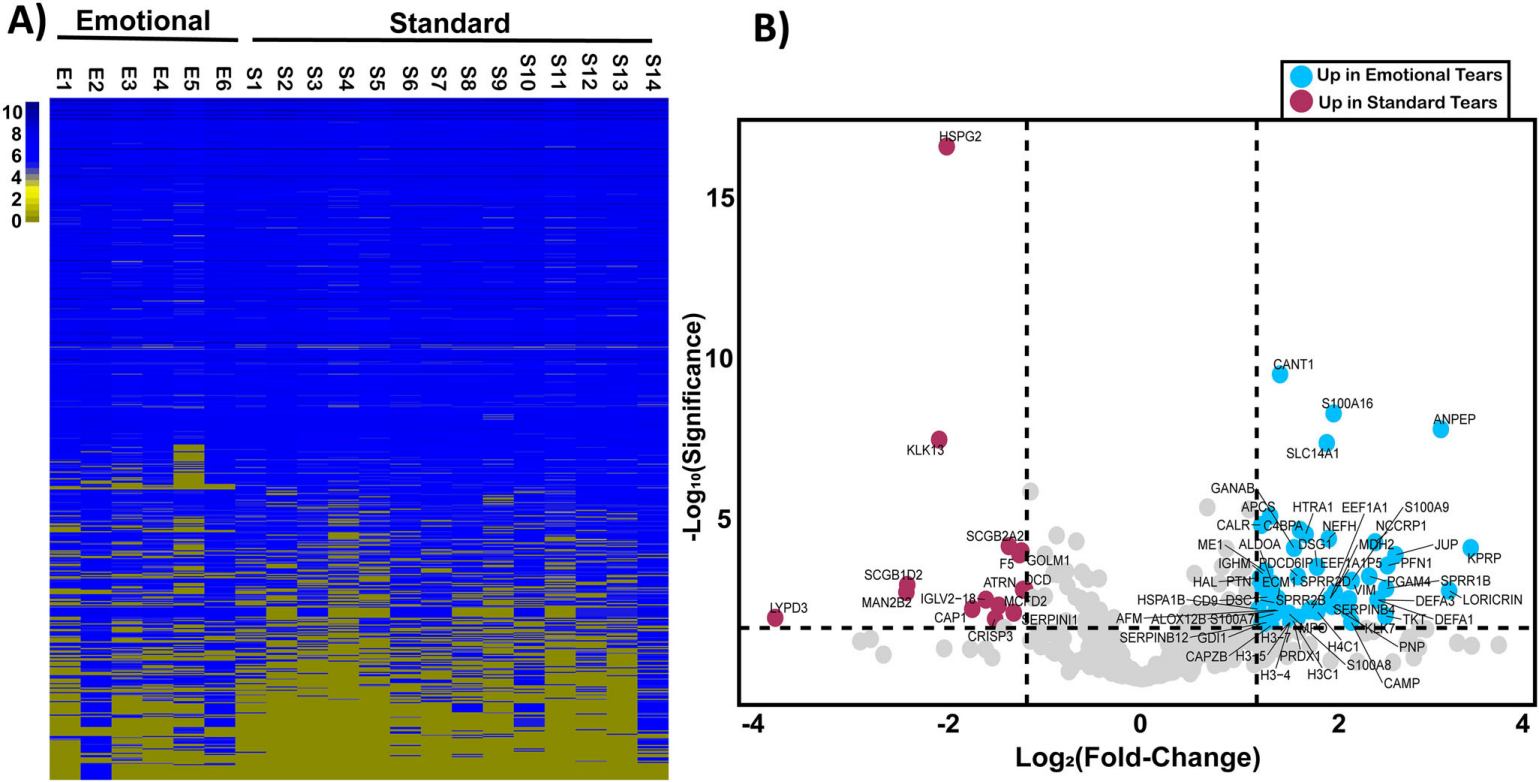
(A) A heat map of all proteins identified in Emotional (E1‐6) and Standard (S1‐14) samples ordered by decreasing average LFQ abundance (Log_10_). Each column represents the average of three technical replicates for a biological replicate. More abundant proteins (log_10_(a.u.) > 10^5^) are shaded blue (darker blue indicates higher LFQ abundance), while less abundant proteins (log_10_(a.u.) < 10^5^) appear a shade of yellow. (B) A volcano plot of fold‐change emotional/standard tears. The *y*‐axis represents significance values as the −log_10_ of *p* values from a Student's *t* test, corrected with the Benjamini‐Hochberg (B‐H) method (FDR of 0.1). The *x*‐axis represents the log_2_ of fold change (LFC), calculated by dividing the average protein LFQ abundance in emotional tears by that in standard tears (emotional [LFQ]/standard [LFQ]). A horizontal dashed line at −log_10_(B‐H *p* value) = 1.57 marks the significance threshold, and vertical dashed lines represent an LFC value greater than 1.2 (log_2_ = |1.2|). Values surpassing these thresholds are considered significant. Significantly enriched proteins are colored blue (emtotional) or red (sta, and proteins that are not significantly changed are gray. Most proteins are unchanged, but there are 64 significantly changed proteins in total: 52 in emotional tears and 12 in standard tears. FDR, false discovery rate; LFQ, label‐free quantification.

Next, proteins were arranged in ascending order based on the log_10_ value of their average LFQ abundances to visualize as a heatmap (Figure 5A). The data highlight the biological variation in protein abundance, particularly among less abundant proteins. The coefficient of variation (CV) was calculated for technical and biological replicates and visualized as box and whisker plots. The average CV of technical replicates is 0.154 (±0.115) for emotional tear samples and 0.136 (±0.103) for standard tears (Figure S3A). However, for biological replicates, these values increase significantly, with average CVs mostly above 0.5 for both tear types (Figure S3B). This is likely due to donor variability, agreeing with previous studies that tears, similar to other biofluids like urine, exhibit a relatively high degree of variation between individuals [43, 44].

### 3.4 Identifying Significantly Enriched Proteins and GO Enrichment Analysis

For enrichment analysis, proteins not identified in at least half of all biological replicates with two or more unique peptides were excluded. LFC and significance values were calculated for qualifying proteins using abundance values. To explore the origin of potential differences in recovered protein abundance between emotional and standard tears, the data were visualized using a volcano plot (Figure 5B). A horizontal dashed line at −log_10_(B‐H *p* value) = 1.57 marks the significance threshold, and vertical dashed lines represent an LFC value greater than 1.2 (log_2_ = |1.2|). Sixty‐four proteins met this criteria for significant enrichment. Specifically, 52 proteins were significantly enriched in emotional tears (blue), and 12 were enriched in standard tears (red), but most of the qualifying proteins identified (500) remain statistically unchanged (gray).

To identify functional associations among the significantly enriched proteins, enriched protein accession and gene IDs were analyzed using the STRING (STRINGdb, v12.0) [35, 45]. STRING analysis of emotional tear proteins revealed significant enrichment of 5 biological processes, 6 molecular functions, and 23 cellular components (Gene Ontology [GO]). In contrast, no functional enrichment was observed for significantly enriched proteins in standard tears. Protein–protein interaction (PPI) network analysis of emotional tear proteins indicated significantly more interactions than expected by chance (*p* < 1.0E‐16), suggesting biological and functional interconnectivity [46].

**FIGURE 6:**
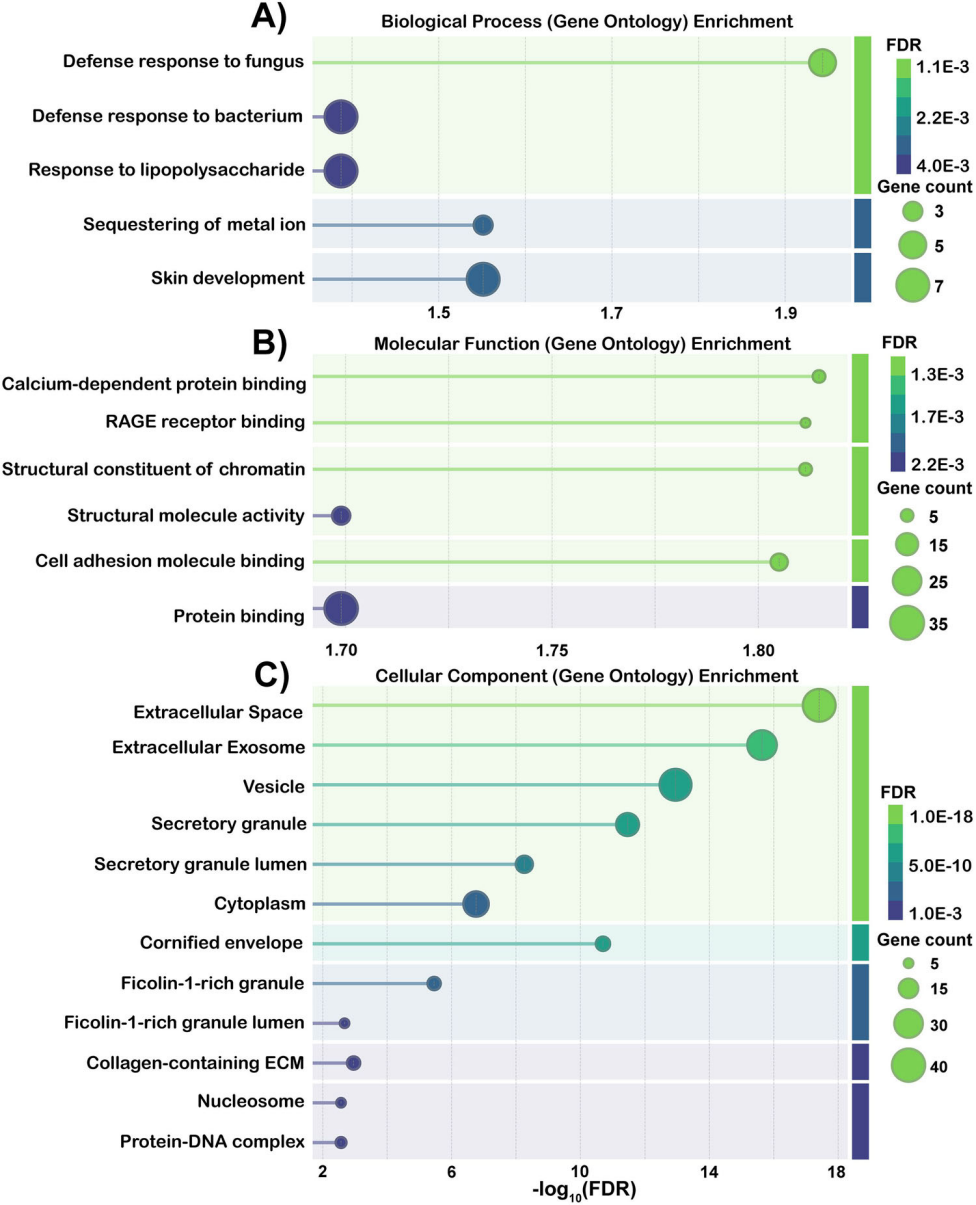
STRING‐db Gene Ontology (GO) enrichment analysis of significantly enriched (LFC > 1.2) proteins in emotional tears. Bubble graphs are used to visualize the significance (−log_10_(FDR)) and count of enriched (A) biological processes, (B) molecular functions, and (C) cellular components. The *y*‐axis represents GO terms, and the *x*‐axis shows significance (−log_10_ FDR). The bubble sizes increase corresponding to gene count, and change in color depending on FDR. FDR, false discovery rate; LFC, Log_2_(fold change); STRING, Search Tool for the Retrieval of Interacting Genes/Proteins.

Data was visualized as a bubble chart (Figure 6), where the *x*‐axis represents the enrichment score (−log_10_(FDR)), the *y*‐axis lists functionally enriched GO terms, and bubble size corresponds to the number of significantly enriched proteins in each category. Bubble color indicates FDR values, and terms are grouped by similarity (≥0.5). The most significantly enriched biological processes in emotional tears were related to defense and immune responses (Figure 6A), while all enriched molecular functions involved binding (Figure 6B). Specifically, the defense response to fungus and sequestering of metal ions exhibited the strongest enrichment signals. RAGE receptor binding, calcium‐dependent protein binding, and cell adhesion molecule binding were the most prominent molecular functions. Three proteins, S100A7, S100A8, and S100A9, were connected to each of these molecular functions and biological processes. S100A8 and S100A9 are primarily found as the calprotectin complex (S100A8/A9), which is known for its broad antimicrobial activity due to its metal‐binding capabilities and its abundance at ocular infection sites [47, 48]. Furthermore, the calprotectin complex, expressed and released by neutrophils, acts as an endogenous ligand for toll‐like receptor 4 and the receptor for advanced glycation end products (RAGE). Based on this information, it came as no surprise that metal sequestration by antimicrobial proteins and neutrophil degranulation were identified as enriched reactome pathways (Figure S4).

The 12 most significantly enriched cellular components (lowest FDR values) were predominantly localized in extracellular, secretory, or cytoplasmic compartments, with a notable representation of extracellular vesicles (EVs) and exosomes (Figure 6C). This distribution is consistent with the secretory nature of tear proteins and highlights the clinical relevance of small extracellular vesicles (sEVs) and exosomes, which play crucial roles in intercellular communication and show promise as therapeutic vehicles [49, 50, 51]. Vesicle markers, including TSG101, CD9, and CD63, have been identified in human tears [52]. Given that exosomal cargo frequently serves as disease biomarkers, there is growing interest in developing technologies capable of detecting and isolating exosomes from microliter‐scale tear samples, which could enhance diagnostic capabilities for ocular and systemic diseases [53, 54]. Other significantly enriched cellular components were ficolin‐1‐rich granules, which are expressed mainly by immune cells (e.g., neutrophils) [55].

## 4 Discussion

Human tears have become valuable targets for biomarker discovery and biosensor development, largely because they are easily accessible and less complex than other biofluids, like serum or plasma [56]. Tear‐based biomarker studies have expanded beyond just ocular diseases, often focusing on detecting alterations in the tear proteome from systemic diseases like cancer, diabetes, and Alzheimer's [57, 58, 59]. However, compositional analysis of all tear types is incomplete, hindering the development of robust and reliable tear‐based diagnostic tools for these and other conditions. Psycho‐emotional tears, specifically, have not been studied as much as other tear types, which limits their potential use in clinical studies [21]. Proteomic analysis of emotional tears could offer more comprehensive insights into tear composition, helping to identify potential biomarkers for personalized medicine and to advance the development of wearable biosensors. In this pilot study, we aimed to expand our functional and compositional understanding of the tear film after emotional stimulation by analyzing the proteome of tears collected from subjects through emotional stimulation (emotional, *n* = 6) versus conventional means (standard, *n* = 14) using shotgun proteomics and LFQ (Figure 1).

Mass spectrometric analysis of the six emotional and fourteen standard tear samples resulted in a total of 907 proteins and 4863 peptides identified (Figure 2A). With a roughly 90% overlap in protein IDs between samples, and similar average numbers of proteins and peptides per sample, the tear protein composition appears relatively consistent (Figure 2B). This consistency is further highlighted by the conserved presence of the 20 most abundant proteins for both tear types (Table S4). Additionally, when comparing the average protein abundances in emotional and standard tears using a scatterplot, we observed a strong linear relationship (PCC = 0.941) (Figure 3B). This indicates a high degree of similarity in protein expression levels between the two tear types. Comparing the class and function of proteins identified in this study to those previously published by Ponzini et al. and Tse et al. revealed a nearly identical distribution of class hits (Figure 4, Figure S2) [41, 42]. These figures confirm that our results align with previously published studies and suggest that the core functional proteins in tears are robustly conserved regardless of stimulation type.

Because there was little variation in protein content between samples, we hypothesized that differentially expressed proteins might reveal specific cellular pathways or functions not directly reflected by overall protein levels. To explore the origin of potential differences in recovered protein abundance between emotional and standard tears, the data was visualized using a volcano plot, which highlights proteins with statistically significant differences in expression and provides insight into their relative abundance changes. This analysis identified 64 statistically enriched proteins across all samples (Figure 5B). Of these statistically enriched proteins, 52 were significantly enriched in emotional tears, while 12 were enriched in standard tears. The accession information from enriched and unique proteins was exported to text for bioinformatic analysis to classify these proteins according to functional associations (Table S5).

The STRING database was used to collect and explore the functional associations of all significantly enriched proteins from these tear samples, aiming to enhance our understanding of the functionality of emotional tears [60]. Paired with the GO tool, this analysis revealed significant enrichment of 5 biological processes, 6 molecular functions, and 23 cellular components (FDR < 0.05) in significantly enriched proteins from emotional tears (Tables S6–S8) [45, 61]. There were no biological processes or molecular functions enriched in emotional tears, and the only functionally enriched biological process was extracellular space. PPI network analysis also revealed that enriched proteins in emotional tears interact with each other more frequently than expected by random chance (*p* < 1.0E‐16), indicating a functional interconnectivity. This is much more significant than the PPI enrichment value generated for standard tears (3.21E‐4). The functional enrichment data was visualized as bubble graphs (Figure 6).

Functionally enriched biological processes were primarily defense responses to fungi and bacteria (or components of bacteria like lipopolysaccharide [LPS]), while enriched molecular functions were binding mechanisms to initiate these responses (Figure 6A, B). Many of the functionally enriched components of emotional tears are linked to the innate immune system as released antimicrobial substances by immune cells through the process of neutrophil degranulation (Figure S4) [62]. Several of the significantly enriched proteins in emotional tears are classified as antimicrobial peptides (AMPs), named for their immunomodulatory and antimicrobial effects. Four members of the S100 protein family (S100A7, A8, A9, A16) were found to be enriched in emotional tears. These proteins are AMPs that bind to calcium and zinc to initiate defense and immune responses [63]. S100A8 and S100A9 make up the calprotectin complex, which has many intra‐ and extracellular functions that inhibit microbial activity [64]. Calprotectin has also demonstrated growth‐inhibitory and apoptosis‐inducing effects on various cell types, including tumor cells and normal fibroblasts, significantly impacting the survival and growth states of cells involved in inflammation [65]. S100A8 and S100A9 were also found to be overexpressed in tears and saliva collected from patients with forms of cancer, but to a much greater extent (∼10‐fold enrichment) than observed in this study [8, 9].

Psoriasin (S100A7) is proven to be highly potent against *Escherichia coli* and other gram‐negative bacteria [66]. It is also shown to be overexpressed in patients with an inflamed skin condition called Psoriasis [67]. Psoriasin and other members of the S100A protein family have been identified as potential drug targets for controlling inflammatory responses. Human neutrophil peptide (HNP) defensin alpha‐1 and ‐2 are other examples of enriched proteins with antibacterial and fungicidal properties. These proteins are naturally present in low levels of basal and reflex tears [68]. Interestingly, HNP ɑ‐defensins and S100 calcium‐binding proteins are found to be upregulated in tear fluid from patients with pterygium, indicating these proteins may also respond to fibrovascular tissue, or just the accompanying inflammation [69].

Enriched cellular components in emotional tears were largely classified as extracellular or secretory, with many classified as vesicles and exosomes. Among the proteins enriched in or unique to emotional tears, 10 are among the most commonly reported biomarkers for sEVs by Vesiclepedia and Exocarta [70, 71]. The most common exosome biomarkers (e.g., CD9, CD63, and TSG101) are identified in both tear types, but only CD9 is enriched in emotional tears. In total, 65 of the Top 100 vesicle‐biomarkers reported by Vesiclepedia or Exocarta are identified in these tear samples (Table S9). Small EVs are secreted from nearly all cell types and are present in biofluids as important intercellular communication regulators with the ability to influence the functions of the cells that receive them [72]. Recent studies have linked sEVs, like exosomes, to ocular and systemic diseases, and have developed methods to isolate them from tears to noninvasively screen for breast cancer and Sjögren's syndrome [54, 73]. These results raise the possibility that tear‐derived sEVs could be utilized to monitor the emotional state of subjects or as diagnostic markers for systemic or ocular conditions related to emotional stress. Other enriched components were ficolin‐1‐rich granules, which are involved in the innate immune system and commonly expressed by neutrophils. Expectedly, neutrophil degranulation and the innate immune system were both enriched reactome in emotional tears (Figure S4).

This work explores the differences in the proteome of tears stimulated by emotion compared to those collected using standard protocols. In this study, we were limited by the number of samples available through vendors, and we are also missing some relevant background data from subjects. Additionally, the use of vendor‐sourced tear samples introduces concerns about sample integrity and handling conditions, as the lack of control over pre‐analytical factors can lead to variability in protein composition. Because of this and the absence of intra‐individual pairing, these findings are exploratory rather than directly clinically applicable and require further validation with larger cohorts. Regardless, a more complete understanding of all tear types is necessary for the continuation of biomarker discovery and wearable biosensor development.

Preliminary results indicate that emotional tears could be a rich source of antimicrobial proteins that play key roles in the innate immune system and neutrophil degranulation, like HNP defensins and S100 calcium‐binding proteins. The functional enrichment of ficolin‐1‐rich granules also implies increased innate immune response activity, as their presence and enrichment are often associated with responses to infection or inflammation. Many of these proteins have been indicated as biomarkers for ocular and other systemic diseases. Additionally, many of the functionally enriched components in emotionally stimulated tears were found to be sEVs and exosomes, which are of clinical interest as both biomarkers and drug delivery vehicles. Further studies with larger cohorts are needed to validate these results. A future, larger‐scale approach could include exosome isolation and comprehensive proteomic and transcriptomic profiling to fully characterize their molecular cargo compared to other tear types.

## Conflicts of Interest

The authors declare no conflicts of interest.

## Open Research

The data that support the findings of this study are openly available in MassIVE at doi: https://doi.org/10.25345/C5W37M74S, reference number MSV00097189.

## Supporting information




**Supporting File 1**: prca70023‐sup‐0001‐TablesS1‐S9.xlsx

**Supporting File 2**: prca70023‐sup‐0002‐FiguresS1‐S4.docx

## Data Availability

The study includes original experimental data deposited in a database.
