# Supplementary material for: A Comparison of Emotionally Stimulated and Conventionally Collected Tears Using Bottom‐Up, Label‐Free Quantitative Proteomic Analysis—A Pilot Study
Source: Proteomics Clin Appl. 2025 Sep 18;20(1):e70023. doi: 10.1002/prca.70023 (PMC12743588; doi:10.1002/prca.70023)
Supplement: Supplementary file 2 — Supporting File 2: prca70023‐sup‐0002‐FiguresS1‐S4.docx [file PRCA-20-e70023-s001.docx]

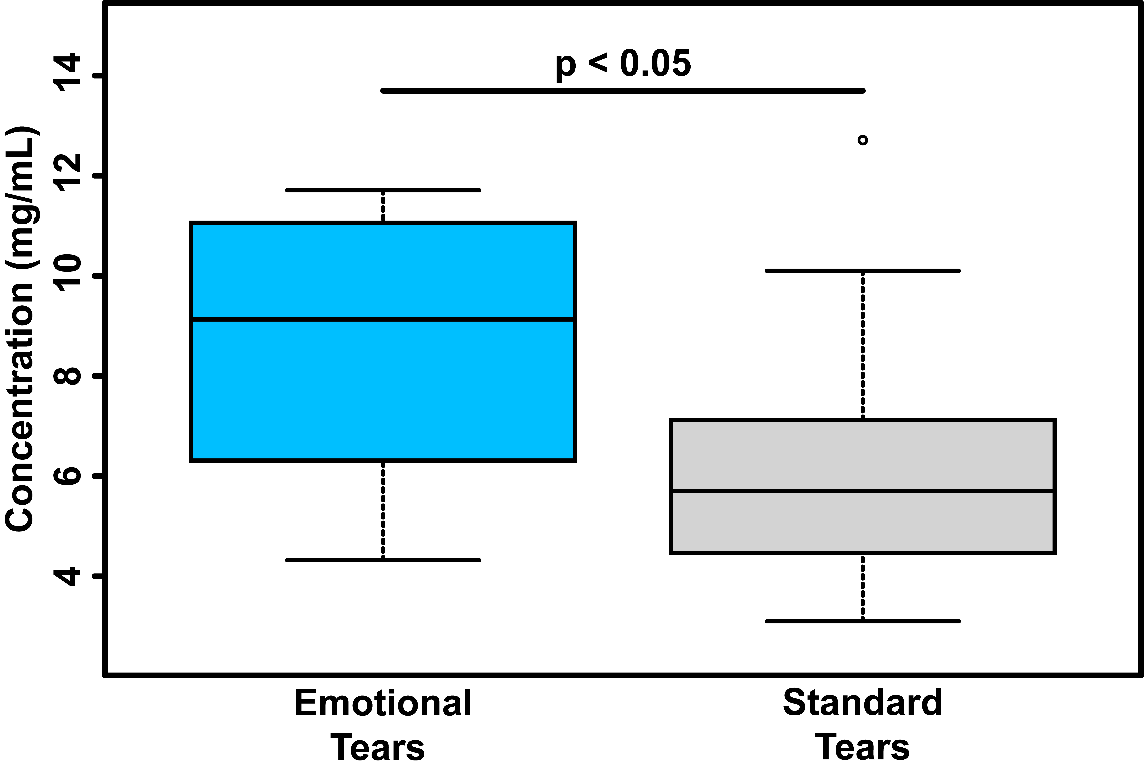


**Figure S1**: Box and whisker plots of average protein concentrations for emotional (blue) and standard (gray) tear samples.


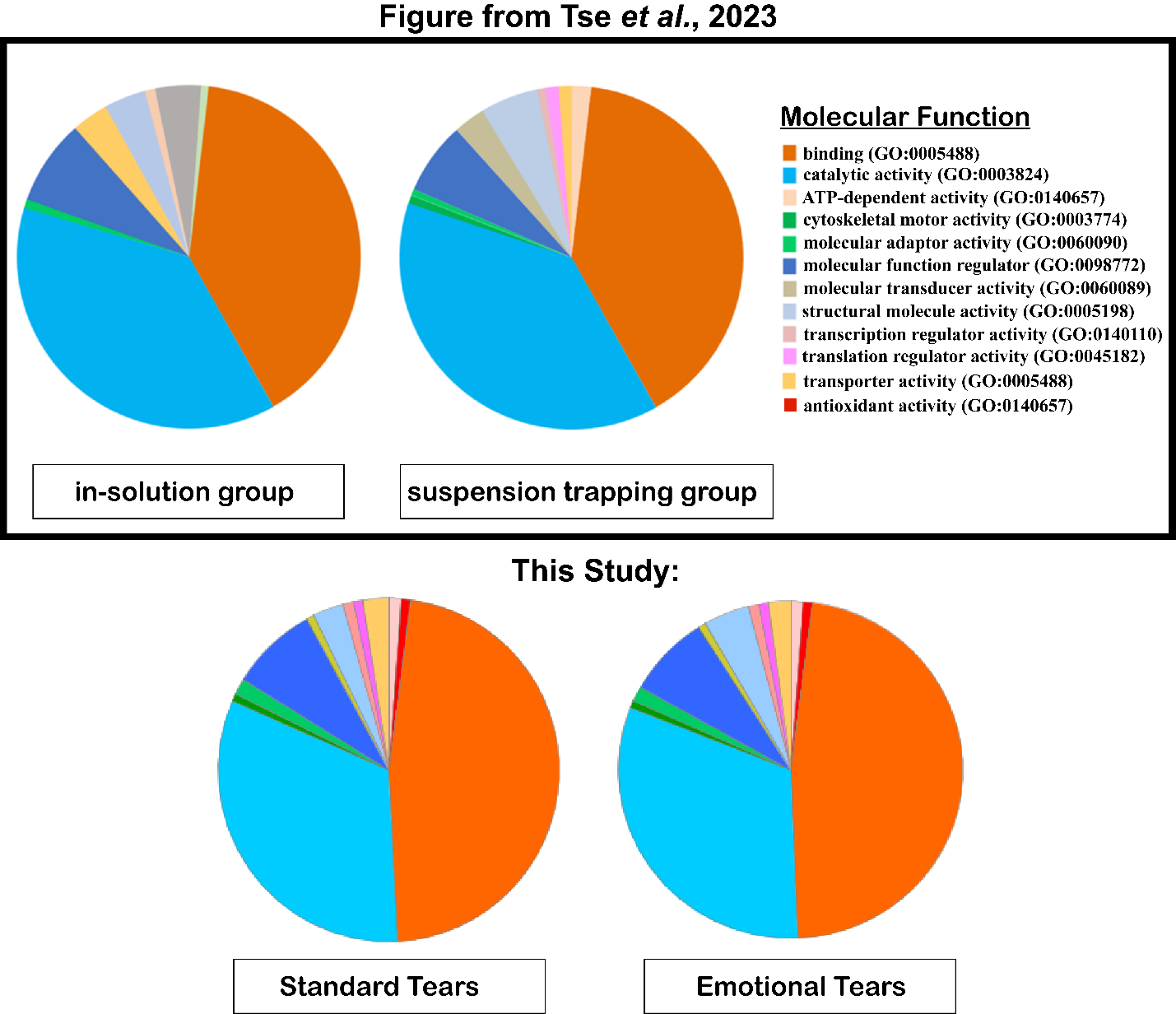


**Figure S2**: Pie charts illustrating the molecular functions of identified tear proteins from in-solution and S-Trap digests from a previously published study (top) and emotional or standard tears from this study (bottom) using the PANTHER classification system.


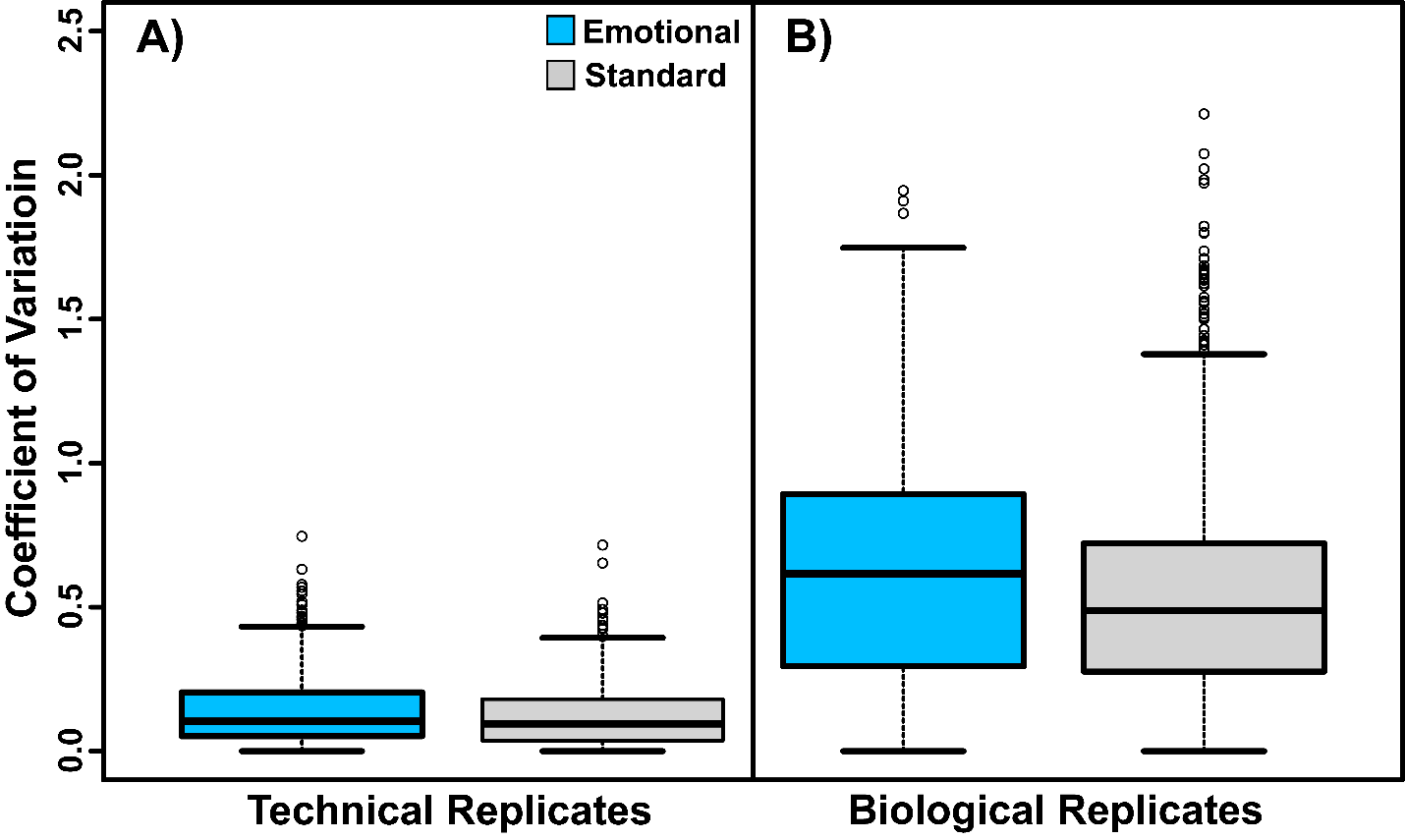


**Figure S3**: Box and whisker plots of average coefficient of variation (CVs) values for protein LFQ abundances from (**A**) technical replicates and (**B**) biological replicates.


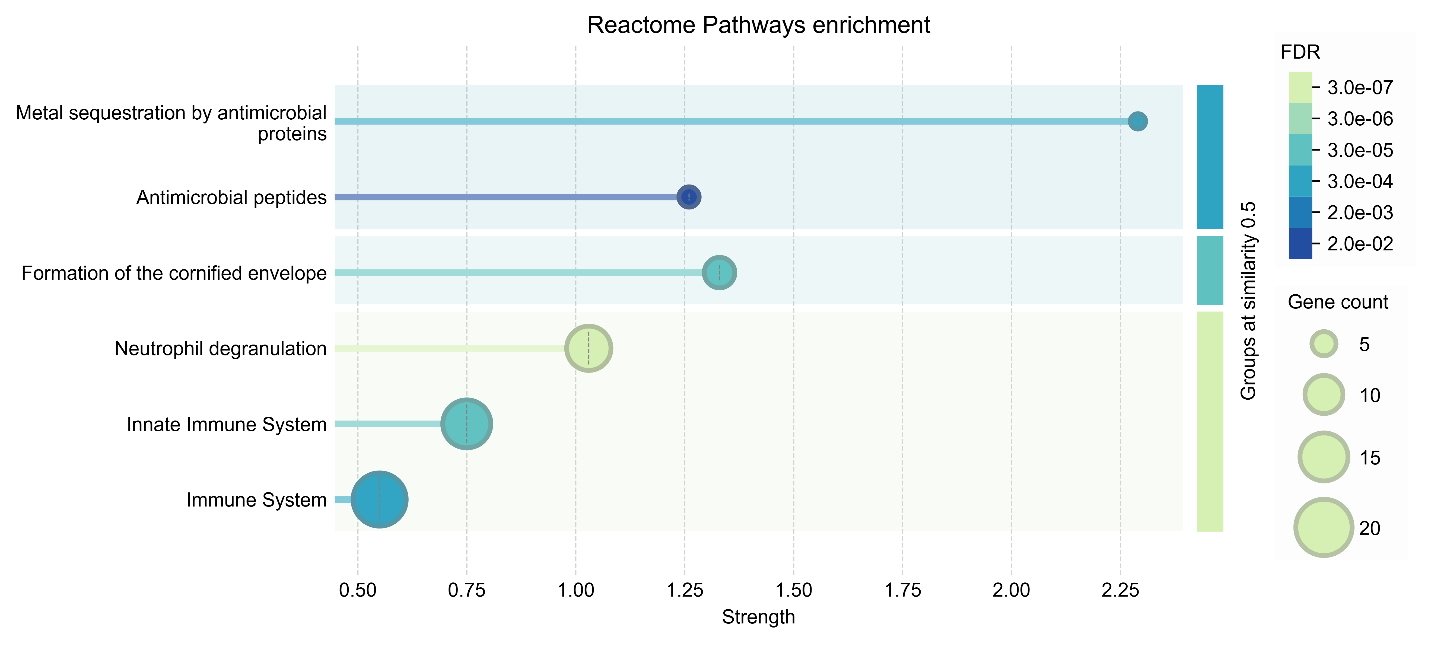


**Figure S4**: Enriched reactome pathways for significantly enriched proteins in emotional tears (STRING-db).
